# A healthy lifestyle pattern and mortality risk in patients with type 2 diabetes mellitus: A prospective cohort study in China

**DOI:** 10.7555/JBR.39.20250107

**Published:** 2025-05-28

**Authors:** Hao Yu, Xinyi Liu, Xueni Cheng, Xikang Fan, Yuefan Shen, Ke Liu, Yanan Wan, Jian Su, Yu Qin, Zhongming Sun, Yan Lu, Shujun Gu, Chong Shen, Dong Hang, Jinyi Zhou

**Affiliations:** 1 Department of Non-communicable Chronic Disease Control, Jiangsu Provincial Center for Disease Control and Prevention, Nanjing, Jiangsu 210009, China; 2 Department of Epidemiology, Jiangsu Key Lab of Cancer Biomarkers, Prevention and Treatment, Collaborative Innovation Center for Cancer Personalized Medicine, School of Public Health, Nanjing Medical University, Nanjing, Jiangsu 211166, China; 3 Department of Epidemiology and Health Statistics, School of Public Health, Southeast University, Nanjing, Jiangsu 210009, China; 4 Department of Chronic Disease Prevention and Control, Huai'an City Center for Disease Control and Prevention, Huai'an, Jiangsu 223001, China; 5 Department of Chronic Disease Prevention and Control, Suzhou City Center for Disease Control and Prevention, Suzhou, Jiangsu 215003, China; 6 Department of Chronic Disease Prevention and Control, Changshu City Center for Disease Control and Prevention, Changshu, Jiangsu 215500, China; 7 Changzhou Medical Center, Nanjing Medical University, Changzhou, Jiangsu 213000, China

**Keywords:** healthy lifestyle, mortality, type 2 diabetes mellitus, prospective cohort study

## Abstract

This study aimed to evaluate the association of a healthy lifestyle pattern with mortality risk among patients with type 2 diabetes mellitus (T2DM). Data were derived from a prospective cohort study enrolling 13776 Chinese patients with T2DM. A healthy lifestyle pattern was constructed based on six lifestyle factors, including smoking status, alcohol consumption, dietary habits, physical activity, sedentary time, and sleep duration. Multivariate Cox proportional hazards models were used to estimate hazard ratios (HRs) and 95% confidence intervals (CIs) for all-cause and cause-specific mortality. During a median follow-up of 9.78 years, 2497 deaths were recorded. Compared with T2DM patients with a lifestyle pattern scoring 0–2, those scoring 5–6 had a 40% lower risk for all-cause mortality (HR = 0.60, 95% CI: 0.52–0.69), a 33% lower risk for cardiovascular disease mortality (HR = 0.67, 95% CI: 0.52–0.86), and a 25% lower risk for cancer mortality (HR = 0.75, 95% CI: 0.58–0.97). Additionally, we found that the association between the lifestyle pattern and all-cause mortality risk was stronger in females than in males (*P* for interaction < 0.05). In conclusion, adherence to a healthy lifestyle pattern is associated with a decreased risk of all-cause, cardiovascular disease, and cancer mortality. These findings have important implications for reducing premature mortality among patients with T2DM.

## Introduction

Type 2 diabetes mellitus (T2DM) represents a pervasive public health challenge globally, with its disease burden steadily escalating over the past few decades^[1]^. Alarmingly, in 2021, approximately 537 million adults aged 20 to 79 were diagnosed with diabetes worldwide, while 6.7 million adults succumbed to the disease, translating to approximately one death every five seconds^[2]^. Projections indicate that the global economic burden of diabetes and its complications will substantially increase to $2.5 trillion by 2030^[3]^. The data emphasize the critical need for intensified efforts in the prevention and effective management of diabetes-related adverse outcomes^[4]^.

In addition to glycemic control^[5]^, lifestyle modification is a fundamental component of diabetes self-management^[6]^. Adopting healthy lifestyle behaviors, such as refraining from smoking, limiting alcohol consumption, maintaining a healthy diet, and engaging in regular physical activity, has been consistently linked to a reduced risk of mortality among individuals with T2DM^[6–9]^. Recently, other emerging factors, such as sleep and sedentary behavior, have attracted widespread attention. The UK Biobank study revealed a "U-shaped" relationship between sleep duration and cardiovascular disease (CVD) mortality^[10]^, while a meta-analysis encompassing 18 studies underscored the association between excessive sedentary time and an elevated all-cause mortality risk^[11]^. However, the evidence base examining the overall influence of a healthy lifestyle on mortality risk in diabetic patients remains limited, and additional efforts are also needed to investigate the influence of sleep duration and sedentary behavior. Furthermore, prior research in diabetic patients has predominantly focused on the association between a healthy lifestyle and total mortality, leaving gaps in knowledge regarding the cause-specific risks of CVD, cancer, and chronic respiratory diseases.

Therefore, leveraging data from a prospective cohort of 13776 individuals with T2DM in China, we investigated the association between a combination of six healthy lifestyle factors and both all-cause and cause-specific mortality in the T2DM population. We aimed to provide valuable insights into the role of holistic lifestyle modifications in reducing mortality risks among T2DM patients.

## Subjects and methods

### Study population

The data were derived from a prospective cohort study named "Comprehensive Research on the Prevention and Control of Diabetes (CRPCD)" in Jiangsu Province, China. Detailed information about the cohort has been described in previous studies^[12]^. After excluding individuals with poor physical or mental status and unqualified questionnaires, a total of 20053 subjects with T2DM completed the investigation during 2013–2014. All participants signed a written statement of informed consent. The current study excluded 4395 participants with cancer, CVD, or chronic respiratory disease at baseline, and 1882 participants with missing data on healthy lifestyle factors (***Supplementary Fig. 1***, available online). Finally, 13776 participants were included in the analysis. Ethical approval was obtained from the Jiangsu Provincial Center for Disease Control and Prevention (No. 2013026).

### Assessment of lifestyle factors

In the present study, we considered six modifiable behavioral factors: smoking, alcohol consumption, physical activity, diet, sleep duration, and sedentary behavior. Details of the assessment for each lifestyle factor are shown in ***Supplementary Table 1*** (available online). Each of the six healthy lifestyle factors was dichotomized into healthy and unhealthy groups. The healthy group for alcohol consumption was defined as non-excessive drinking, which included those who never drank, formerly drank, or drank moderately^[13]^. The healthy group of nonsmokers comprised never smokers or former smokers who had quit for reasons other than illness^[14]^. For physical activity, the healthy group was defined as those with levels above the median, after accounting for age (< 50, 50–59, and ≥ 60 years) and sex differences^[15]^. For dietary habits, the healthy group was defined as those who consumed at least four recommended food categories and met the recommended frequency of consumption. For sleep duration, 7–8 h per day was considered adequate^[16]^. For sedentary behavior, the healthy group was defined as no more than 4 h per day of inactivity^[17]^. For each factor, a low risk (healthy) level was assigned 1 point, and an unhealthy level was assigned 0 points. The overall lifestyle score was calculated as the sum of the six factors, ranging from zero to six, with a higher score indicating better adherence to a healthy lifestyle. To avoid extreme groups with limited participants, the lifestyle score was subsequently divided into four categories (0–2, 3, 4, and 5–6). Because of possible weight fluctuations caused by T2DM, body mass index (BMI) was not included in the lifestyle score to minimize reverse causation bias^[18]^.

### Assessment of covariates

Covariates were obtained from the baseline questionnaire, including sociodemographic characteristics, self-reported diseases, and medication use. Diabetes duration was calculated by subtracting the age at T2DM diagnosis from the baseline age^[19]^. BMI was calculated by dividing weight in kilograms by the square of height in meters. Glycated hemoglobin (HbA1c) was determined by high-performance liquid chromatography (Burroughs D-10 glycosylated hemoglobin meter)^[20]^.

### Ascertainment of outcomes

Death-related information was obtained from the provincial death registration system of the Jiangsu Center for Disease Control and Prevention (JSCDC). Each case of death was collected by qualified staff and medically verified by senior personnel to ensure its completeness and accuracy. All causes of death were coded according to the International Classification of Diseases, 10th Revision (ICD-10) (***Supplementary Table 2***, available online). In the current study, the main outcomes included all-cause (A00-Y89), CVD (I00-I99), cancer (C00-C97), and chronic respiratory disease (J30-J98) mortality. Participants were followed up from the date of baseline questionnaire completion to the date of death or the censoring date (September 30, 2023), whichever occurred first. Person-years were calculated by dividing the follow-up time by 365.25^[21]^.

### Statistical analysis

Baseline characteristics were presented as medians (interquartile range, IQR) for continuous variables and as percentages for categorical variables. Nonparametric tests were used to compare continuous variables between groups. Unordered categorical variables were compared using the Chi-square test, while ordered categorical variables were compared using nonparametric tests. The Cox proportional hazards model was used to calculate hazard ratios (HRs) and 95% confidence intervals (CIs) for the association between healthy lifestyle factors and all-cause and cause-specific mortality. The lifestyle score was treated as a continuous variable to test the linear trend. When analyzing individual lifestyle factors, all five other lifestyle factors were adjusted simultaneously. We constructed two multivariate adjustment models to account for potential confounders. Model 1 was adjusted for age (years) at baseline and sex (male, female). Model 2 was further adjusted for education level (uneducated, primary school, middle school, high school and above, unknown), annual household income (< 10, 10–39.9, 40–99.9, ≥ 100 thousand yuan, unknown), BMI (kg/m^2^), waist circumference (centimeters), hypertension (yes, no, unknown), dyslipidemia (yes, no, unknown), diabetes duration (years), oral antidiabetic medication use (yes, no, unknown), and insulin use (yes, no, unknown). We categorized healthy lifestyle factors into two categories (0–4 *vs*. 5–6 factors) and calculated population-attributable risk (PAR) to estimate the proportion of all-cause and cause-specific mortality that could theoretically be prevented if all participants adhered to 5–6 healthy lifestyle factors.

Stratified analyses were performed by age (< 65, ≥ 65 years), sex (male, female), BMI (< 24, ≥ 24 kg/m^2^), diabetes duration (< 5, ≥ 5 years), oral antidiabetic medication use (yes, no), insulin use (yes, no), and HbA1c level (< 7.0%, ≥ 7.0% [53 mmol/mol]). To test for heterogeneity, likelihood ratio tests were used to compare models with and without interaction (cross-product) terms between the lifestyle score and stratification variables.

Several sensitivity analyses were conducted to assess the robustness of the results. First, we excluded participants who died within the first two years of follow-up (*n* = 275). Second, we constructed a weighted lifestyle score considering the strength of the association between each lifestyle factor and mortality risk. Third, we performed cause-specific mortality analysis using the competing risk proportional subdistribution hazards regression model to account for the presence of competing events.

All analyses were performed using R (version 4.2.2; The R Foundation for Statistical Computing, Vienna, Austria). A two-sided *P*-value < 0.05 was considered statistically significant.

## Results

### Population characteristics

Baseline characteristics of the study population are presented in ***Table 1***. Of 13776 participants with T2DM at baseline, the median baseline age was 62 years (IQR: 56–69 years), and 8421 (61.1%) were females. The proportion of those scoring 0–2, 3, 4, and 5–6 was 16.4%, 27.9%, 33.4%, and 22.3%, respectively. Compared with participants in the lowest score group (0–2), those in the highest score group (5–6) were younger, had smaller waist circumference, and were less likely to have hypertension or dyslipidemia. No significant differences were observed across groups for diabetes duration, oral antidiabetic medication use, or insulin therapy (all *P* > 0.05).

**Table 1:** Baseline characteristics of participants with type 2 diabetes by healthy lifestyle score category

Characteristics	Total	Healthy lifestyle score				*P*-value
		0–2 (*n*=2258)	3 (*n*=3840)	4 (*n*=4601)	5–6 (*n*=3077)	
Age at baseline (years)	62.0 (56.0, 69.0)	63.0 (56.0, 70.0)	63.0 (56.0, 70.0)	62.0 (56.0, 68.0)	61.0 (56.0, 67.0)	<0.001
Sex						<0.001
Male	5355 (38.9)	1497 (66.3)	1534 (39.9)	1443 (31.4)	881 (28.6)	
Female	8421 (61.1)	761 (33.7)	2306 (60.1)	3158 (68.6)	2196 (71.4)	
Education^ a^						<0.001
Uneducated	4814 (34.9)	671 (29.7)	1449 (37.7)	1724 (37.5)	970 (31.5)	
Primary school	4881 (35.4)	849 (37.6)	1326 (34.5)	1633 (35.5)	1073 (34.9)	
Middle school	2711 (19.7)	482 (21.3)	699 (18.2)	872 (19.0)	658 (21.4)	
High school and above	1329 (9.6)	251 (11.1)	352 (9.2)	358 (7.8)	368 (12.0)	
Annual household income (thousand yuan)^a^						<0.001
<10	1843 (13.4)	354 (15.7)	591 (15.4)	613 (13.3)	285 (9.3)	
10–39.9	3406 (24.7)	470 (20.8)	961 (25.0)	1164 (25.3)	811 (26.4)	
40–99.9	6385 (46.3)	1010 (44.7)	1739 (45.3)	2129 (46.3)	1507 (49.0)	
≥100	2070 (15.0)	416 (18.4)	521 (13.6)	676 (14.7)	457 (14.9)	
Hypertension^a^						<0.001
Yes	7236 (52.5)	1277 (56.6)	2075 (54.0)	2365 (51.4)	1519 (49.4)	
No	6329 (45.9)	943 (41.8)	1709 (44.5)	2159 (46.9)	1518 (49.3)	
Dyslipidemia^a^						<0.001
Yes	2007 (14.6)	392 (17.4)	562 (14.6)	613 (13.3)	440 (14.3)	
No	9569 (69.5)	1457 (64.5)	2670 (69.5)	3236 (70.3)	2206 (71.7)	
BMI (kg/m^2^)	25.0 (22.9, 27.2)	24.8 (22.8, 27.1)	25.1 (23.0, 27.2)	24.9 (22.9, 27.1)	25.0 (23.0, 27.2)	0.045
Waist circumference (cm)	86.0 (80.0, 92.0)	87.0 (81.0, 93.0)	86.0 (80.0, 92.2)	85.0 (79.0, 91.5)	85.0 (79.3, 91.0)	<0.001
Diabetes duration (years)	4.0 (2.0, 8.0)	4.0 (2.0, 9.0)	4.0 (2.0, 8.0)	4.0 (2.0, 9.0)	5.0 (2.0, 8.0)	0.874
Oral antidiabetic medication use^a^						0.175
Yes	9427 (68.4)	1549 (68.6)	2650 (69.0)	3174 (69.0)	2054 (66.8)	
No	4247 (30.8)	699 (31.0)	1157 (30.1)	1393 (30.3)	998 (32.4)	
Insulin use^a^						0.244
Yes	1965 (14.3)	337 (14.9)	575 (15.0)	631 (13.7)	422 (13.7)	
No	11709 (85.0)	1911 (84.6)	3232 (84.2)	3936 (85.5)	2630 (85.5)	
Data are presented as median (interquartile range) and *n* (%) for continuous and categorical variables, respectively.^a^For some variables, the totals do not sum to 100% due to the small proportion of participants choosing "prefer not to answer".Abbreviation: BMI, body mass index.						

### All-cause mortality

**Figure 1:**
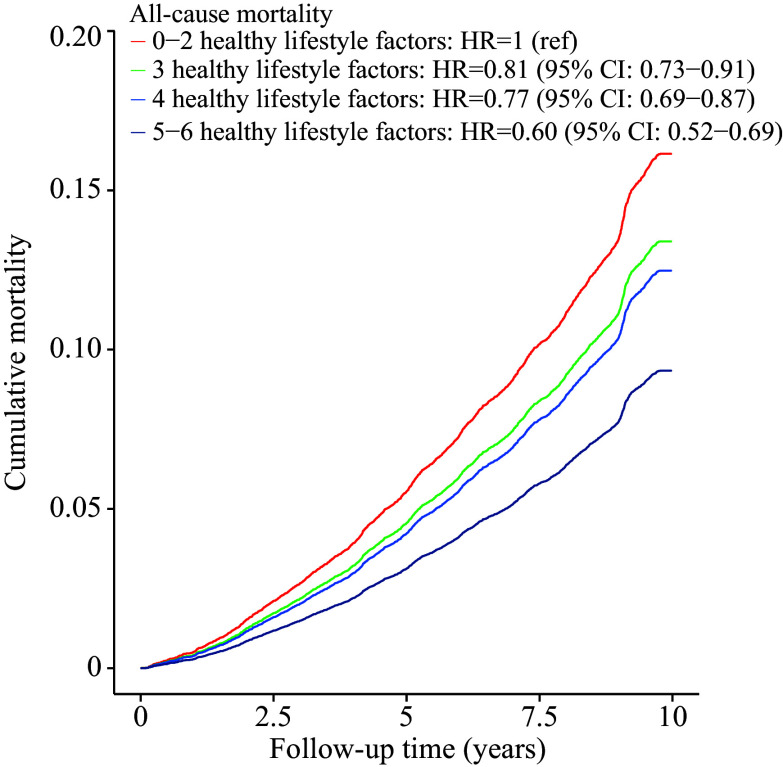
Effect of combined healthy lifestyles on all-cause mortality. Participants were divided into four groups based on lifestyle score (0–2, 3, 4, and 5–6). HRs were calculated in Cox proportional hazards model, adjusted for age at baseline (years), sex (male or female), education level (uneducated, primary school, middle school, high school and above, unknown), annual household income (< 10, 10–39.9, 40–99.9, ≥ 100 thousand yuan, unknown), BMI (kg/m^2^), waist circumference (cm), hypertension (yes, no, unknown), dyslipidemia (yes, no, unknown), diabetes duration (years), oral antidiabetic medication use (yes, no, unknown), and insulin use (yes, no, unknown). Abbreviations: BMI, body mass index; CI, confidence interval; HR, hazard ratio.

During a median follow-up of 9.78 years (IQR: 9.70–9.82), 2497 deaths were recorded, with 751 (30.08%) from CVD, 618 (24.75%) from cancer, and 151 (6.05%) from chronic respiratory disease. A higher lifestyle score was associated with a lower risk of all-cause mortality (HR per 1-point increase = 0.88, 95% CI: 0.84–0.91) (***Table 2***). Compared with those scoring 0–2, T2DM patients scoring 3, 4, and 5–6 had HRs of 0.81 (95% CI: 0.73–0.91), 0.77 (95% CI: 0.69–0.87), and 0.60 (95% CI: 0.52–0.69), respectively (***Fig. 1***). Compared with participants scoring 5–6, those scoring 0–4 had PAR for all-cause mortality of 23.50% (95% CI: 17.50%–29.50%) at the median follow-up (***Table 2***). When analyzing individual lifestyle factors, we found that healthy dietary habits, adequate sleep, regular physical activity, and less sedentary time were associated with reduced risks of all-cause mortality in model 1. The associations of healthy dietary habits (HR = 0.78, 95% CI: 0.71–0.85), regular physical activity (HR = 0.76, 95% CI: 0.70–0.83), and reduced sedentary behavior (HR = 0.90, 95% CI: 0.83–0.98) with the risk of all-cause mortality remained statistically significant in model 2 (***Supplementary Table 3***, available online).

**Table 2:** HR (95% CI) of all-cause and cause-specific mortality according to combined healthy lifestyles

Cause of mortality	Healthy lifestyle score^a^				*P* for trend^b^	HR (95% CI) per 1 score point^c^	PAR% (95% CI)^d^
	0–2	3	4	5–6			
All-cause							
No. of cases/person-years	588/19738	776/34341	767/42043	366/28718			
Model 1	1	0.81 (0.72−0.90)	0.76 (0.68−0.84)	0.55 (0.49−0.63)	<0.001	0.86 (0.83−0.89)	23.64 (17.63−29.65)
Model 2	1	0.81 (0.73−0.91)	0.77 (0.69−0.87)	0.60 (0.52−0.69)	<0.001	0.88 (0.84−0.91)	23.50 (17.50−29.50)
CVD							
No. of cases/person-years	165/19738	250/34341	225/42043	111/28718			
Model 1	1	0.92 (0.75−1.12)	0.80 (0.65−0.99)	0.62 (0.48−0.79)	<0.001	0.88 (0.82−0.94)	24.30 (12.10−36.51)
Model 2	1	0.92 (0.75−1.12)	0.82 (0.67−1.01)	0.67 (0.52−0.86)	0.001	0.90 (0.84−0.96)	24.30 (12.10−36.50)
Cancer							
No. of cases/person-years	141/19738	166/34341	197/42043	114/28718			
Model 1	1	0.76 (0.61−0.95)	0.83 (0.66−1.03)	0.72 (0.56−0.93)	0.024	0.92 (0.86−0.99)	10.93 (0−25.36)
Model 2	1	0.77 (0.61−0.96)	0.84 (0.67−1.05)	0.75 (0.58−0.97)	0.051	0.93 (0.87−1.00)	9.92 (0−24.51)
Chronic respiratory disease							
No. of cases/person-years	29/19738	47/34341	59/42043	16/28718			
Model 1	1	1.03 (0.65−1.64)	1.32 (0.84−2.07)	0.58 (0.31−1.08)	0.175	0.91 (0.79−1.05)	43.25 (16.96−69.54)
Model 2	1	1.11 (0.69−1.77)	1.44 (0.91−2.28)	0.73 (0.39−1.36)	0.548	0.96 (0.82−1.11)	42.11 (15.36−68.87)
Other							
No. of cases/person-years	253/19738	313/34341	286/42043	125/28718			
Model 1	1	0.73 (0.62−0.87)	0.63 (0.53−0.75)	0.42 (0.34−0.53)	<0.001	0.80 (0.75−0.84)	35.38 (25.50−45.27)
Model 2	1	0.73 (0.62−0.87)	0.65 (0.55−0.77)	0.46 (0.37−0.58)	<0.001	0.82 (0.77−0.87)	35.58 (25.78−45.38)
^a^Healthy lifestyle factors were defined as non-smoking (never smokers or smoking cessation for reasons other than illness); non-excessive alcohol consumption (never drank, formerly drank or drank moderately); healthy dietary habits (adequate intake of at least one-half of eight recommended food categories); regular physical activity (above median levels after accounting for age [< 50 years, 50–59 years, and ≥ 60 years] and sex differences); adequate sleep duration (7–8 h/day) and less sedentary time (< 4 h/day). ^b^*P* for trend and per lifestyle factors were calculated by a continuous variable of healthy lifestyle factors. ^c^HRs were calculated in the Cox proportional hazards model. Model 1 was adjusted for age at baseline (years) and sex (male or female). Model 2 was further adjusted for education level (uneducated, primary school, middle school, high school and above, unknown), annual household income (< 10, 10–39.9, 40–99.9, ≥ 100 thousand yuan, unknown), body mass index (kg/m^2^), waist circumference (cm), hypertension (yes, no, unknown), dyslipidemia (yes, no, unknown), diabetes duration (years), oral antidiabetic medication use (yes, no, unknown) and insulin use (yes, no, unknown).^d^The percentage of all-cause and cause-specific mortality theoretically attributable to non-adherence to 5–6 healthy lifestyle factors among participants included in the current study. PAR at the median follow-up time of the study population was reported. The population-attributable risk for CVD mortality associated with healthy lifestyle factors calculated was negative and therefore was considered to be zero.Abbreviations: CI, confidence interval; CVD, cardiovascular disease; HR, hazard ratio; PAR, population-attributable risk.							

### Cause-specific mortality

The lifestyle score was inversely associated with mortality from CVD, cancer, and other causes, whereas the association with chronic respiratory disease mortality was not statistically significant. Compared with the group with a healthy lifestyle score of 0–2, those with a score of 5–6 had HRs of 0.67 (95% CI: 0.52–0.86) for CVD mortality, 0.75 (95% CI: 0.58–0.97) for cancer mortality, 0.46 (95% CI: 0.37–0.58) for mortality from other causes, and 0.73 (95% CI: 0.39–1.36) for chronic respiratory disease mortality. When analyzing individual lifestyle factors, we found that participants adhering to healthy dietary habits (HR = 0.74; 95% CI: 0.62–0.88) and regular physical activity (HR = 0.80; 95% CI: 0.69–0.93) had a lower risk of cardiovascular disease mortality (***Supplementary Table 4***, available online). Compared with current smokers, former or never smokers had a lower risk of cancer mortality (HR = 0.78; 95% CI: 0.64-0.95) (***Supplementary Table 5***, available online). The PAR for participants scoring 0–4 was 24.30% (95% CI: 12.10%–36.50%) for CVD mortality, 9.92% (95% CI: 0%–24.51%) for cancer mortality, and 42.11% (95% CI: 15.36%–68.57%) for chronic respiratory disease mortality (***Table 2***). In the subtype analysis of CVD and cancer, the results showed that compared with participants scoring 0–2, those scoring 5–6 had a lower mortality risk from stroke and esophageal cancer (***Supplementary Fig. 2***, available online).

### Stratified and sensitivity analyses

**Figure 2:**
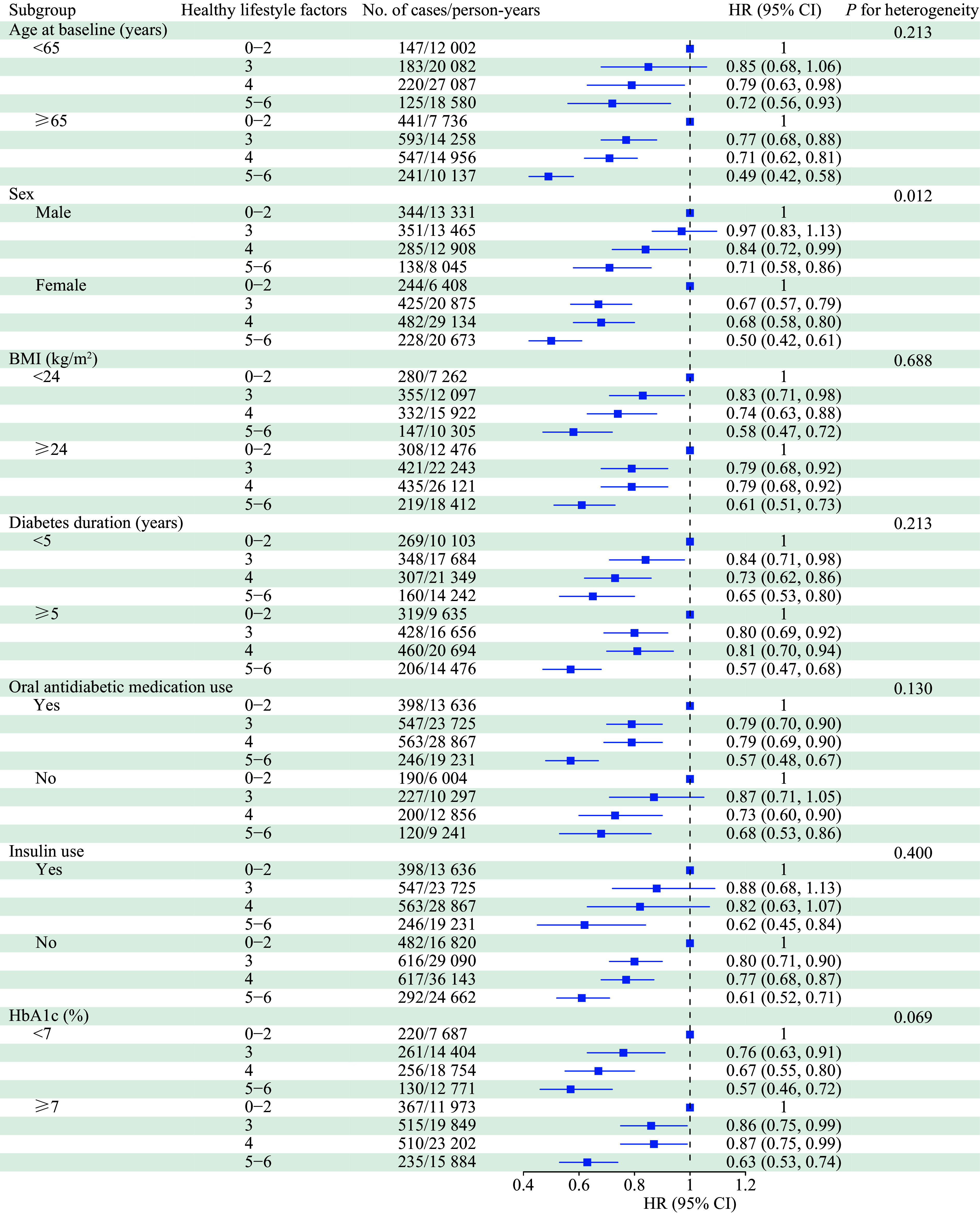
Association between lifestyle score and all-cause mortality risk stratified by potential risk factors. HRs were calculated in Cox proportional hazards model, adjusted for age at baseline (years), sex (male or female), education level (uneducated, primary school, middle school, high school and above, unknown), annual household income (< 10, 10–39.9, 40–99.9, ≥ 100 thousand yuan, unknown), BMI (kg/m^2^), waist circumference (cm), hypertension (yes, no, unknown), dyslipidemia (yes, no, unknown), diabetes duration (years), oral antidiabetic medication use (yes, no, unknown), and insulin use (yes, no, unknown). The stratification variable was not included in the model when stratifying by itself. Abbreviations: BMI, body mass index; CI, confidence interval; HbA1c, glycated hemoglobin A1c; HR, hazard ratio.

The analysis was stratified by age, sex, BMI, diabetes duration, oral antidiabetic medication use, insulin use, and HbA1c level. The associations were generally consistent across the subgroups, except for the stratification by sex. The association between a combined healthy lifestyle and all-cause mortality risk was stronger in females than in males (*P* for heterogeneity = 0.012; ***Fig. 2***).

In the sensitivity analysis excluding participants who died within the first two years of follow-up, the association between lifestyle score and cancer mortality became marginally significant in the fully adjusted model, whereas the other associations remained largely unchanged (***Supplementary Table 6***, available online). The results also remained stable when the weighted lifestyle score was applied (***Supplementary Table 7***, available online) or when competing risks for cause-specific mortality were taken into account (***Supplementary Table 8***, available online).

## Discussion

In this cohort study involving 13776 participants with T2DM, we examined the association between adherence to a combination of six healthy lifestyle factors and both all-cause and cause-specific mortality. Our results demonstrate that adherence to a healthy lifestyle significantly reduces the risk of all-cause mortality, as well as mortality because of CVD and cancer.

A comprehensive meta-analysis of 16 cohort studies showed that adhering to a healthy lifestyle significantly reduced all-cause mortality by 56% among patients with T2DM^[22]^. Notably, the majority of these studies were conducted in the United States and Europe, with only one study conducted in Asia. Moreover, there are inconsistencies in the definition of a healthy lifestyle among these studies, and few have included factors such as sleep duration or sedentary behavior, which are particularly relevant to the Chinese population. Our study contributes to the existing evidence by not only incorporating these additional factors but also expanding the study outcomes to encompass several major chronic diseases, thereby providing novel insights into the association between healthy lifestyle factors and mortality among T2DM patients.

CVD and cancer are the major causes of death among individuals with T2DM^[23–24]^. A prospective cohort study indicated that adherence to a composite healthy lifestyle, encompassing a high-quality diet, non-smoking, moderate-to-vigorous physical activity, and moderate alcohol consumption, was associated with a lower risk of CVD mortality in adults with T2DM^[6]^. Meanwhile, an international cohort study of 376354 individuals with diabetes revealed an inverse association between a healthy lifestyle (current non-smoking, low-to-moderate alcohol drinking, adequate physical activity, healthy diet, and optimal body weight) and cancer mortality^[25]^. Our study further expands upon these findings by incorporating sleep duration and sedentary time into the healthy lifestyle pattern, reinforcing the protective effect of this comprehensive lifestyle paradigm on mortality from both CVD and cancer. Diabetes can contribute to CVD and carcinogenesis through various mechanisms, including hyperinsulinemia, hyperglycemia, dysregulation of insulin-like growth factor, and chronic inflammation^[26]^. Conversely, a healthy lifestyle may mitigate these risks by reducing pro-inflammatory stimuli, oxidative stress, hormonal imbalances, and abnormal methylation, ultimately lowering the risk of CVD and cancer mortality among T2DM patients^[27]^.

Sedentary behavior has emerged as an independent risk factor for mortality among patients with T2DM^[28]^, yet the lack of standardization in its definition and measurement methods across studies remains a challenge. In the current study, we defined sedentary behavior as self-reported sitting, leaning, or lying down, and designated < 4 h per day as the healthy group, based on previous literature^[29]^. Our findings suggest that a higher level of sedentary behavior is associated with an increased all-cause mortality risk among individuals with T2DM. This aligns with a meta-analysis of the general population, which reported a 49% and 90% increase in the risk of all-cause and CVD mortality, respectively, with longer sedentary time^[11]^. Furthermore, a prospective cohort study observed elevated levels of triglycerides, glucose, and indices of insulin resistance among individuals with prolonged sedentary time, suggesting that extended sedentary periods may be detrimental to glycemic control^[30]^.

The strengths of this prospective study include its large sample size comprising T2DM patients from the community, coupled with an extended follow-up period. Furthermore, the rich database facilitated nuanced analyses of specific mortality and allowed for stratification based on potential risk factors, enhancing statistical rigor. However, several limitations should be noted. First, information was mainly collected *via* participant self-report; therefore, recall bias cannot be ruled out. Second, data on healthy lifestyle behaviors were collected only at baseline, and changes or long-term patterns may not have been captured through a single assessment. Finally, although several confounding factors were adjusted for during the analysis, the influence of other unmeasured confounders cannot be entirely excluded.

In conclusion, our prospective cohort study underscores the inverse association between adopting a comprehensive healthy lifestyle, encompassing non-excessive alcohol consumption, non-smoking, a healthful dietary pattern, regular physical activity, sufficient sleep duration, and minimal sedentary time, and decreased mortality among patients with T2DM. This finding holds considerable importance for reducing the risk of premature death associated with T2DM and highlights the critical role of lifestyle modifications in the management and prevention of diabetes-related complications.

## SUPPLEMENTARY DATA

Supplementary data to this article can be found online.
