# Supplementary material for: A healthy lifestyle pattern and mortality risk in patients with type 2 diabetes mellitus: A prospective cohort study in China
Source: J Biomed Res. 2025 May 28;39(6):639–47. doi: 10.7555/JBR.39.20250107 (PMC12683505; doi:10.7555/JBR.39.20250107)
Supplement: Supplementary file 1 — Supplementary data to this article can be found online. [file jbr-39-6-639-Supplementary.pdf]

Available online at www.jbr-pub.org.cn

Open Access at PubMed Central

Journal of Biomedical Research, 2025 39(6): 1–7

JBR

Supplementary Data

A healthy lifestyle pattern and mortality risk in patients with type 2 diabetes mellitus: A prospective cohort study in China

Hao Yu[1,△], Xinyi Liu[2,△], Xueni Cheng[2], Xikang Fan[1], Yuefan Shen[2], Ke Liu[3], Yanan Wan[1], Jian Su[1], Yu Qin[1], Zhongming Sun[4], Yan Lu[5], Shujun Gu[6], Chong Shen[2], Dong Hang[2,7,✉], Jinyi Zhou[1,✉]

[1]*Department of Non-communicable Chronic Disease Control, Jiangsu Provincial Center for Disease Control and Prevention, Nanjing, Jiangsu 210009, China;*
[2]*Department of Epidemiology, Jiangsu Key Lab of Cancer Biomarkers, Prevention and Treatment, Collaborative Innovation Center for Cancer Personalized Medicine, School of Public Health, Nanjing Medical University, Nanjing, Jiangsu 211166, China;*
[3]*Department of Epidemiology and Health Statistics, School of Public Health, Southeast University, Nanjing, Jiangsu 210009, China;*
[4]*Department of Chronic Disease Prevention and Control, Huai'an City Center for Disease Control and Prevention, Huai'an, Jiangsu 223001, China;*
[5]*Department of Chronic Disease Prevention and Control, Suzhou City Center for Disease Control and Prevention, Suzhou, Jiangsu 215003, China;*
[6]*Department of Chronic Disease Prevention and Control, Changshu City Center for Disease Control and Prevention, Changshu, Jiangsu 215500, China;*
[7]*Changzhou Medical Center, Nanjing Medical University, Changzhou, Jiangsu 213000, China.*

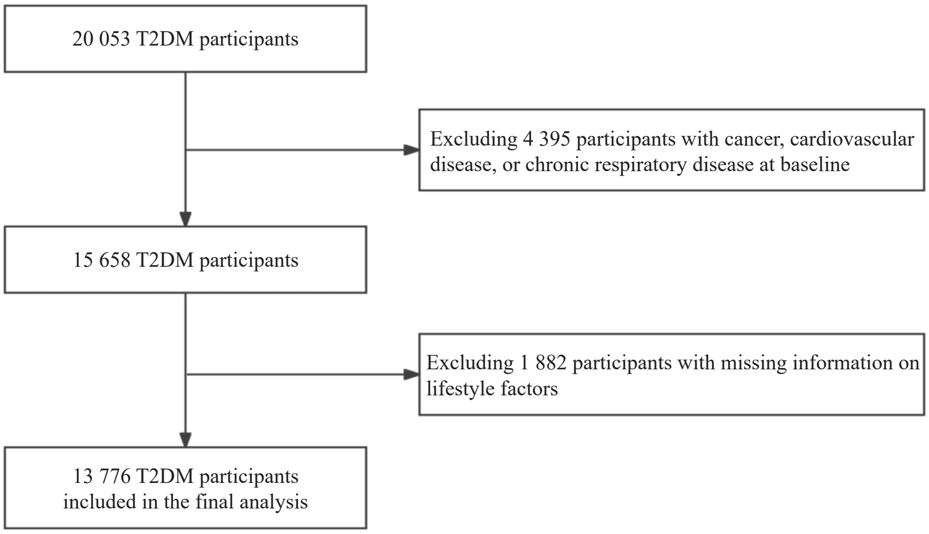

Supplementary Fig. 1 **Flowchart of participants included in the analysis.** Abbreviation: T2DM, type 2 diabetes mellitus.

△These authors contributed equally to this work.

✉Corresponding authors: Jinyi Zhou, Jiangsu Provincial Center for Disease Control and Prevention, 172 Jiangsu Road, Nanjing, Jiangsu 210009, China. E-mail: zhoujinyi74@sina.com; Dong Hang, Department of Epidemiology, School of Public Health, Nanjing Medical University, 101 Longmian Avenue, Nanjing, Jiangsu 211166, China. E-mail: hangdong@njmu.edu.cn.

Received: 12 March 2025; Revised: 28 April 2025; Accepted: 22 May 2025; Published online: 28 May 2025

CLC number: R587.1, Document code: A

The authors reported no conflict of interests.

This is an open access article under the Creative Commons Attribution (CC BY 4.0) license, which permits others to distribute, remix, adapt and build upon this work, for commercial use, provided the original work is properly cited.

© 2025 by Journal of Biomedical Research.

https://doi.org/10.7555/JBR.39.20250107

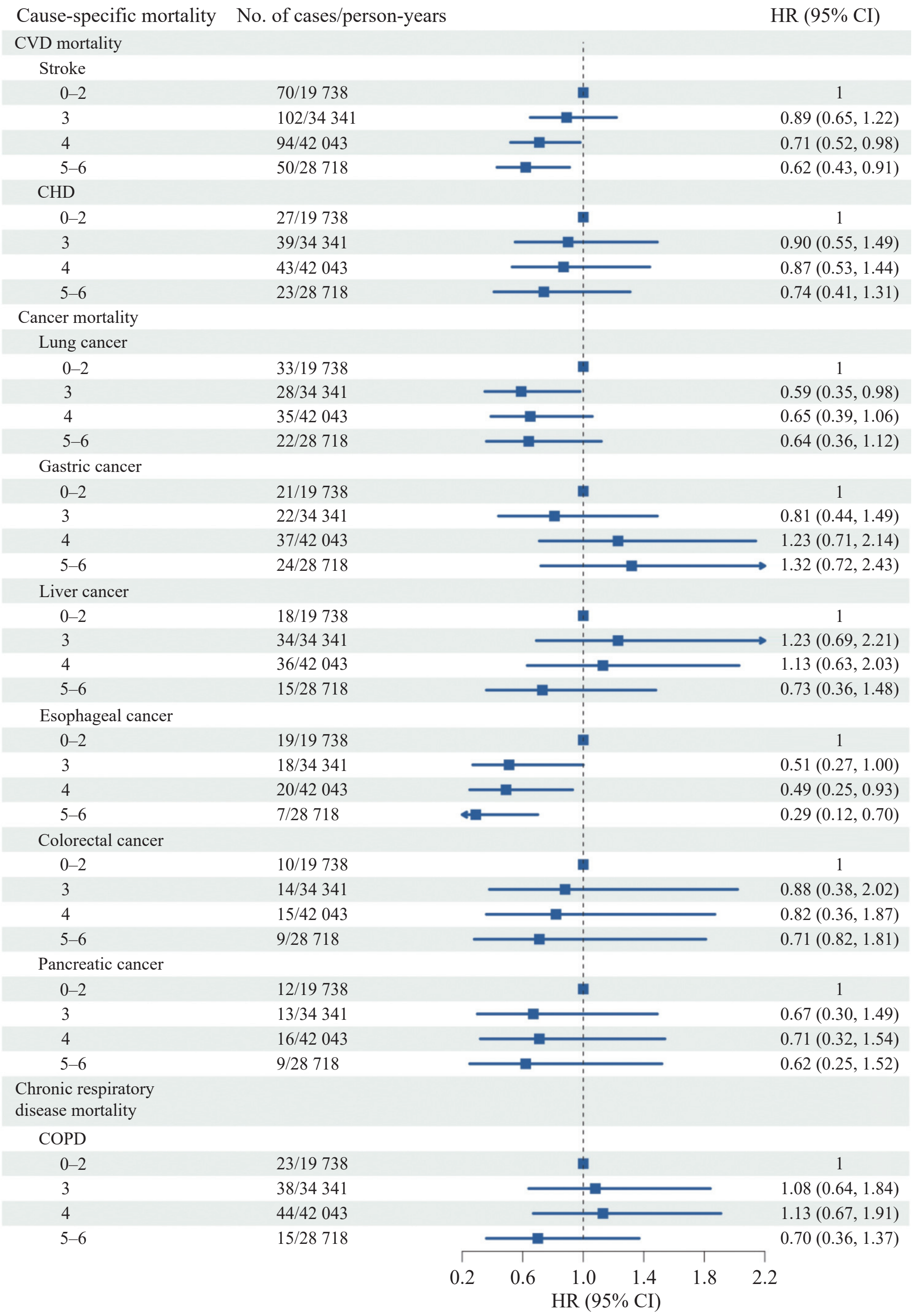

Supplementary Fig. 2 **The associations between lifestyle scores and the risk of cause-specific mortality based on the fully adjusted Cox regression models.** HRs were calculated in the Cox proportional hazards model, adjusted for age at baseline (years), sex (male or female), education level (uneducated, primary school, middle school, high school and above, unknown), annual household income (< 10, 10–39.9, 40–99.9, ⩾ 100 thousand yuan, unknown), body mass index (kg/m^2), waist circumference (cm), hypertension (yes, no, unknown), dyslipidemia (yes, no, unknown), diabetes duration (years), oral antidiabetic medication use (yes, no, unknown) and insulin use (yes, no, unknown). Abbreviations: CI, confidence interval; COPD, chronic obstructive pulmonary disease; HR, hazard ratio.

Supplementary Table 1 **Assessment of lifestyle factors**

Lifestyle factors	Intake goal	Questionnaire
Alcohol consumption	Never drank, previously drank, or drank moderately	Participants were asked about drinking frequency through the following question: "Are you drinking now? (1. Yes, 2. I used to drink but do not drink now, 3. No)", " How often have you been drinking in the past 12 months? (1. Every day, 2. 5–6 days/week, 3. 3–4 days/week, 4. 1–2 days/week, 5. 1–3 days/month, and 6. Less than 1 day/month)". According to the reported drinking frequency, participants were further asked "How much Baijiu (quantity), beer (bottle), yellow rice wine (quantity), and wine (quantity) did you drink on an average day?". We calculated the average alcohol intake of each participant by multiplying the amount of alcohol consumed per day, frequency of alcohol consumption (daily, weekly/7, or monthly/30), and the alcohol content corresponding to different types of alcohol. Overall alcohol consumption was classified into four categories: never, previously, moderate alcohol use (currently drinking with an average alcohol consumption ≤ 15 g/day), and excessive alcohol use (currently drinking with an average alcohol consumption ≥ 15 g/day).
Smoking	Never smokers or smoking cessation for reasons other than illness	Participants were asked about their current smoking status through the following question: "Have you smoked at least 100 cigarettes so far?" (1. Yes, 2. No), "Do you smoke now?" (1. Yes, every day, 2. Yes, but not every day, 3. Used to smoke, but not now). In addition, we also asked, "If you quit smoking and why did you quit?". Then, we divided smoking status into three groups (current, previous, and never).
Dietary habits	≥ 4 recommended food categories	A localized short food frequency questionnaire (FFQ) based on the FFQ in the 2010 Chinese Chronic Disease Noncommunicable and Risk Factor Surveillance was used. According to the Chinese Dietary Guidelines, eight common foods were selected for dietary information assessment in this study, including fresh vegetables, fresh fruits, whole grains, eggs, fish/seafood, dairy products, soybean products, and red meat. For each component, the recommended healthy consumption frequency was as follows: eating fresh vegetables/fresh fruits/whole grains/eggs daily, eating fish/seafood ≥ 2 days per week, eating dairy/soybean products ≥ 4 days per week, and eating red meat 1–6 days per week. Participants were asked about dietary habits through the following questions: "How often have you eaten fresh fruit in the past 12 months? (Fruits refer to all types of unprocessed fresh fruits)", "How often have you eaten fresh vegetables in the past 12 months? (Vegetables refer to all types of unprocessed fresh vegetables.)", "How often have you eaten whole grains in the past 12 months? (Such as millet, sorghum, corn, buckwheat, oats, red beans, mung beans, potatoes, *etc.*)", "How many times a week have you eaten eggs in the past 12 months? (Such as fresh chicken, duck or goose eggs without special processing)", "How often have you eaten fish/seafood in the past 12 months? (Such as fresh or frozen freshwater fish, shrimp, crab, saltwater fish, shrimp, crab, shells, snails, *etc.*)", "How often have you eaten dairy products in the past 12 months? (Such as fresh milk, yogurt, milk powder, cheese, *etc.*)", "How often have you eaten soybean products in the past 12 months? (Such as tofu, soybean milk, *etc.*)", "How often have you eaten red meat in the past 12 months? (Such as unprocessed pork, and domestic animal meat, including cattle, sheep, dogs, *etc.*)". Healthy dietary habits were defined as an adequate intake of at least half of the eight recommended food categories.
Physical activity	Physical activity level above median levels after accounting for age (< 50 years, 50–59 years, and ≥ 60 years) and sex differences	Participants were asked about physical activity equivalent through the following question: "How many days in a week do you usually have moderate or high-intensity physical activities for work, transportation, and leisure?", "How much time do you usually spend on moderate and high-intensity activities for work, transportation, and leisure in a day?". To calculate daily physical activity levels, we multiplied the metabolic equivalent task (MET) value for each type of activity by the number of hours spent on each activity and summed the MET hours for all activities.
Sleep duration (h/day)	7–8	Participants were asked about sleep duration through the following question: "How much time do you sleep in a day? ".
Sedentary behavior (h/day)	< 4	Participants were asked about sedentary behavior through the following question: "How much time do you spend sitting, leaning, or lying down in a day (including the time for sitting, working, studying, reading, watching TV, using the computer, resting and all other static behaviors, but excluding sleeping time)? ".

Supplementary Table 2 **International Classification of Diseases (ICD) codes used for identification of cause-specific mortality**

Cause of death	ICD-10 code	No. of cases
All-cause	A00-Y89	2 497
Cardiovascular disease	I00-I99	751
Coronary heart disease	I20-I25	132
Stroke	I60-I64	316
Cancer	C00-C97	618
Esophageal cancer	C15	64
Gastric cancer	C16	104
Colorectal cancer	C18-C20	48
Liver cancer	C22	103
Pancreatic cancer	C25	50
Lung cancer	C34	118
Chronic respiratory disease	J30-J98	151
Chronic obstructive pulmonary disease	J40-J44	120

Supplementary Table 3 **HR (95% CI) for all-cause mortality by individual healthy lifestyle score**

Healthy lifestyle factors	No. of cases/ person-years	HR (95% CI)[a]	
		Model 1	Model 2
Alcohol consumption			
Non-moderate	222/12 375	1	1
Moderate, previous or never	2 275/112 464	1.08 (0.93–1.25)	1.05 (0.91–1.22)
Smoking			
Current	588/27 748	1	1
Previous or never	1 909/97 091	0.92 (0.83–1.02)	0.95 (0.85–1.06)
Dietary habits			
< 4 recommended food categories	1 895/86 569	1	1
⩾ 4 recommended food categories	602/38 270	0.72 (0.66–0.79)	0.78 (0.71–0.85)
Physical activity			
Non-regular	1 569/63 527	1	1
Regular	928/61 312	0.73 (0.67–0.80)	0.76 (0.70–0.83)
Sleep duration (h/day)			
< 7 or > 8	1 404/62 208	1	1
7–8	1 093/62 631	0.91 (0.84–0.98)	0.96 (0.88–1.04)
Sedentary behavior (h/day)			
⩾ 4	961/41 409	1	1
< 4	1 536/83 430	0.88 (0.81–0.95)	0.90 (0.83–0.98)

[a]HRs were calculated in the Cox proportional hazards model. Model 1 was adjusted for age at baseline (years) and sex (male or female). Model 2 was further adjusted for education level (uneducated, primary school, middle school, high school and above, unknown), annual household income (< 10, 10–39.9, 40–99.9, ⩾ 100 thousand yuan, unknown), body mass index (kg/m^2), waist circumference (cm), hypertension (yes, no, unknown), dyslipidemia (yes, no, unknown), diabetes duration (years), oral antidiabetic medication use (yes, no, unknown) and insulin use (yes, no, unknown). All other five lifestyle factors were simultaneously adjusted for analyses on the association of each individual lifestyle factor with all-cause mortality risk.
Abbreviations: CI, confidence interval; HR, hazard ratio.

Supplementary Table 4 **HR (95% CI) for CVD mortality by individual healthy lifestyle score**

Healthy lifestyle factors	No. of cases/person-years	HR (95% CI)[a]	
		Model 1	Model 2
Alcohol consumption			
Non-moderate	57/12 375	1	1
Moderate, previous or never	694/112 464	1.19 (0.90–1.58)	1.19 (0.89–1.59)
Smoking			
Current	163/27 748	1	1
Previous or never	588/97 091	0.97 (0.80–1.18)	0.97 (0.80–1.19)
Dietary habits			
< 4 recommended food categories	577/86 569	1	1
⩾ 4 recommended food categories	174/38 270	0.69 (0.58–0.81)	0.74 (0.62–0.88)
Physical activity			
Non-regular	471/63 527	1	1
Regular	280/61 312	0.77 (0.66–0.90)	0.80 (0.69–0.93)
Sleep duration (h/day)			
< 7 or > 8	414/62 208	1	1
7–8	337/62 631	0.99 (0.85–1.14)	1.04 (0.90–1.20)
Sedentary behavior (h/day)			
⩾ 4	298/41 409	1	1
< 4	453/83 430	0.86 (0.74–1.00)	0.87 (0.75–1.01)

[a]HRs were calculated in the Cox proportional hazards model. Model 1 was adjusted for age at baseline (years), sex (male or female). Model 2 was further adjusted for education level (uneducated, primary school, middle school, high school and above, unknown), annual household income (< 10, 10–39.9, 40–99.9, ⩾ 100 thousand yuan, unknown), body mass index (kg/m^2), waist circumference (cm), hypertension (yes, no, unknown), dyslipidemia (yes, no, unknown), diabetes duration (years), oral antidiabetic medication use (yes, no, unknown) and insulin use (yes, no, unknown). All five other lifestyle factors were simultaneously adjusted for analyses on the association of each individual lifestyle factor with all-cause mortality risk.
Abbreviations: CI, confidence interval; HR, hazard ratio.

Supplementary Table 5 **HR (95% CI) for cancer mortality by individual healthy lifestyle score**

Healthy lifestyle factors	No. of cases/person-years	HR (95% CI)[a]	
		Model 1	Model 2
Alcohol consumption			
Non-moderate	87/12 375	1	1
Moderate, previous or never	531/112 464	0.76 (0.59–0.97)	0.81 (0.63–1.04)
Smoking			
Current	189/27 748	1	1
Previous or never	429/97 091	0.73 (0.60–0.89)	0.78 (0.64–0.95)
Dietary habits			
< 4 recommended food categories	450/86 569	1	1
⩾ 4 recommended food categories	168/38 270	0.83 (0.70–1.00)	0.86 (0.71–1.03)
Physical activity			
Non-regular	337/63 527	1	1
Regular	281/61 312	0.95 (0.81–1.12)	0.96 (0.81–1.12)
Sleep duration (h/day)			
< 7 or > 8	323/62 208	1	1
7–8	295/62 631	0.99 (0.84–1.16)	1.01 (0.86–1.18)
Sedentary behavior (h/day)			
⩾ 4	199/41 409	1	1
< 4	419/83 430	1.11 (0.94–1.32)	1.11 (0.94–1.32)

[a]HRs were calculated in the Cox proportional hazards model. Model 1 was adjusted for age at baseline (years) and sex (male or female). Model 2 was further adjusted for education level (uneducated, primary school, middle school, high school and above, unknown), annual household income (< 10, 10–39.9, 40–99.9, ⩾ 100 thousand yuan, unknown), body mass index (kg/m^2), waist circumference (cm), hypertension (yes, no, unknown), dyslipidemia (yes, no, unknown), diabetes duration (years), oral antidiabetic medication use (yes, no, unknown) and insulin use (yes, no, unknown). All other five lifestyle factors were simultaneously adjusted for analyses on the association of each individual lifestyle factor with all-cause mortality risk.
Abbreviations: CI, confidence interval; HR, hazard ratio.

Supplementary Table 6 **Sensitivity analyses excluding deaths within the first two years of follow-up**

Cause of mortality	Healthy lifestyle score				*P* for trend[a]	HR (95% CI) per 1 score point[b]
	0–2	3	4	5–6		
All-cause						
No. of cases/person-years	525/19 662	682/34 232	680/41 936	335/28 682		
HR (95% CI)	1	0.79 (0.70–0.88)	0.75 (0.67–0.84)	0.60 (0.52–0.69)	<0.001	0.86 (0.82–0.90)
CVD						
No. of cases/person-years	148/19 662	226/34 232	199/41 936	104/28 682		
HR (95% CI)	1	0.90 (0.74–1.12)	0.79 (0.63–0.98)	0.68 (0.52–0.88)	0.001	0.88 (0.81–0.95)
Cancer						
No. of cases/person-years	126/19 662	140/34 232	169/41 936	106/28 682		
HR (95% CI)	1	0.71 (0.56–0.91)	0.79 (0.62–1.00)	0.76 (0.58–1.00)	0.133	0.94 (0.86–1.02)
Chronic respiratory disease						
No. of cases/person-years	28/19 662	45/34 232	56/41 936	13/28 682		
HR (95% CI)	1	1.07 (0.66–1.73)	1.38 (0.87–2.21)	0.97 (0.30–1.17)	0.622	0.96 (0.81–1.14)
Other						
No. of cases/person-years	223/19 662	271/34 232	256/41 936	112/28 682		
HR (95% CI)	1	0.71 (0.59–0.85)	0.64 (0.53–0.77)	0.45 (0.36–0.58)	<0.001	0.79 (0.74–0.85)

[a]*P* for trend and per score point were calculated by a continuous variable of healthy lifestyle factors.
[b]HRs were calculated in the Cox proportional hazards model, adjusted for age at baseline (years), sex (male or female), education level (uneducated, primary school, middle school, high school and above, unknown), annual household income (< 10, 10–39.9, 40–99.9, ≥ 100 thousand yuan, unknown), body mass index (kg/m^2), waist circumference (cm), hypertension (yes, no, unknown), dyslipidemia (yes, no, unknown), diabetes duration (years), oral antidiabetic medication use (yes, no, unknown) and insulin use (yes, no, unknown).
Abbreviations: CI, confidence interval; CVD, cardiovascular disease; HR, hazard ratio.

Supplementary Table 7 **Association of weighted lifestyle score with all-cause and cause-specific mortality risk**

Cause of mortality	Weighted healthy lifestyle score[a]				*P* for trend[b]	HR (95% CI) per 1 score point[c]
	Q1 (< 0.43)	Q2 (0.43–1.17)	Q3 (1.17–1.68)	Q4 (1.68–2.90)		
All-cause						
No. of cases/person-years	644/20 335	765/34 726	827/49 113	261/20 665		
HR (95% CI)	1	0.78 (0.70–0.87)	0.71 (0.64–0.79)	0.54 (0.46–0.62)	< 0.001	0.83 (0.80–0.87)
CVD						
No. of cases/person-years	183/20 335	252/34 726	248/49 113	68/20 665		
HR (95% CI)	1	0.91 (0.75–1.10)	0.78 (0.64–0.95)	0.52 (0.39–0.69)	< 0.001	0.83 (0.77–0.90)
Cancer						
No. of cases/person-years	131/20 335	161/34 726	236/49 113	90/20 665		
HR (95% CI)	1	0.81 (0.64–1.02)	0.91 (0.73–1.13)	0.79 (0.60–1.03)	0.243	0.95 (0.88–1.03)
Chronic respiratory disease						
No. of cases/person-years	31/20 335	46/34 726	53/49 113	21/20 665		
HR (95% CI)	1	1.10 (0.70–1.75)	1.15 (0.73–1.82)	1.08 (0.61–1.91)	0.682	1.04 (0.88–1.23)
Other						
No. of cases/person-years	299/20 335	306/34 726	290/49 113	82/20 665		
HR (95% CI)	1	0.66 (0.56–0.78)	0.55 (0.46–0.64)	0.38 (0.42–0.48)	< 0.001	0.73 (0.69–0.79)

[a]The weighted lifestyle score was constructed as follows: the binary lifestyle variables were multiplied by the β coefficients, summed up, divided by the sum of β coefficients, and multiplied by 6. We then categorized it into four groups based on the quartile of the weighted lifestyle score: Q1, Q2, Q3, and Q4. A higher level indicates higher adherence to a healthy lifestyle.
[b]*P* for trend and per lifestyle factors were calculated by a continuous variable of healthy lifestyle factors.
[c]HRs were calculated in the Cox proportional hazards model, adjusted for age at baseline (years), sex (male or female), education level (uneducated, primary school, middle school, high school and above, unknown), annual household income (< 10, 10–39.9, 40–99.9, ≥ 100 thousand yuan, unknown), body mass index (kg/m^2), waist circumference (cm), hypertension (yes, no, unknown), dyslipidemia (yes, no, unknown), diabetes duration (years), oral antidiabetic medication use (yes, no, unknown) and insulin use (yes, no, unknown).
Abbreviations: CI, confidence interval; CVD, cardiovascular disease; HR, hazard ratio.

Supplementary Table 8 **Association of health lifestyle score with all-cause and cause-specific mortality risk using competing risk regression**

Cause of mortality	Healthy lifestyle score				*P* for trend[a]	HR (95% CI) per 1 score point[b]
	0–2	3	4	5–6		
CVD						
No. of cases/person-years	165/19 738	250/34 341	225/42 043	111/28 718		
HR (95% CI)	1	0.95 (0.78–1.16)	0.85 (0.69–1.05)	0.68 (0.53–0.87)	0.001	0.89 (0.82–0.95)
Cancer						
No. of cases/person-years	141/19 738	166/34 341	197/42 043	114/28 718		
HR (95% CI)	1	0.79 (0.63–1.00)	0.89 (0.71–1.11)	0.80 (0.62–1.04)	0.240	0.95 (0.88–1.03)
Chronic respiratory disease						
No. of cases/person-years	29/19 738	47/34 341	59/42 043	16/28 718		
HR (95% CI)	1	1.73 (0.99–3.00)	1.10 (0.66–1.83)	1.32 (0.63–2.80)	0.980	1.00 (0.82–1.21)
Other						
No. of cases/person-years	253/19 738	313/34 341	286/42 043	125/28 718		
HR (95% CI)	1	0.75 (0.63–0.89)	0.64 (0.54–0.77)	0.44 (0.35–0.55)	< 0.001	0.78 (0.73–0.83)

[a]*P* for trend and per lifestyle factors were calculated by a continuous variable of healthy lifestyle factors.
[b]HRs were calculated in the Cox proportional hazards model, adjusted for age at baseline (years), sex (male or female), education level (uneducated, primary school, middle school, high school and above, unknown), annual household income (< 10, 10–39.9, 40–99.9, ⩾ 100 thousand yuan, unknown), body mass index (kg/m^2), waist circumference (cm), hypertension (yes, no, unknown), dyslipidemia (yes, no, unknown), diabetes duration (years), oral antidiabetic medication use (yes, no, unknown) and insulin use (yes, no, unknown).
Abbreviations: CI, confidence interval; CVD, cardiovascular disease; HR, hazard ratio.
